# Biomarkers characterization of circulating tumour cells in breast cancer patients

**DOI:** 10.1186/bcr3180

**Published:** 2012-05-03

**Authors:** Rosa Nadal, Ana Fernandez, Pedro Sanchez-Rovira, Marta Salido, María Rodríguez, José Luis García-Puche, Marta Macià, Josep Maria Corominas, Miguel Delgado-Rodriguez, Lucas Gonzalez, Joan Albanell, Mónica Fernández, Francesc Solé, José Antonio Lorente, María José Serrano

**Affiliations:** 1 Molecular Cytogenetics Laboratory, Pathology Department, Parc de Salut Mar-Hospital del Mar- IMIM-GRETNHE, Passeig Marítim, 25-29, Barcelona, ES-08003, Spain; 2 Medicine Department, Universitat Autònoma de Barcelona, Passeig Vall d'Hebrón, 119, Barcelona, ES-08035, Spain; 3 Pfizer-University of Granada-Andalusian Government Center of Genomics and Oncology (GENyO), Avenida de la Ilustración, 114, Granada, ES-18007, Spain; 4 Medical Oncology Department, University Hospital of Jaén, Avenida del Ejército Español, 10, Jaén, ES-23007, Spain; 5 Medical Oncology Department, Hospital del Mar-IMAS, Passeig Marítim, 25-29, Barcelona, ES-08003, Spain; 6 Division of Preventive Medicine and Public Health, CIBERESP, University of Jaén, Campus de las Lagunillas, Ctra Torrequebradilla s/n, Edificio B-3, Jaén, ES-23071, Spain; 7 Laboratory of Genetic Identification-UGR, Department of Legal Medicine, University of Granada, Avenida Madrid, 11, Granada, ES-18012, Spain

## Abstract

**Introduction:**

Increasing evidence supports the view that the detection of circulating tumor cells (CTCs) predicts outcomes of nonmetastatic breast cancer patients. CTCs differ genetically from the primary tumor and may contribute to variations in prognosis and response to therapy. As we start to understand more about the biology of CTCs, we can begin to address how best to treat this form of disease.

**Methods:**

Ninety-eight nonmetastatic breast cancer patients were included in this study. CTCs were isolated by immunomagnetic techniques using magnetic beads labelled with a multi-CK-specific antibody (CK3-11D5) and CTC detection through immunocytochemical methods. Estrogen receptor, progesterone receptor and epidermal growth factor receptor (EGFR) were evaluated by immunofluorescence experiments and HER2 and TOP2A by fluorescence *in situ *hybridization. We aimed to characterize this set of biomarkers in CTCs and correlate it with clinical-pathological characteristics.

**Results:**

Baseline detection rate was 46.9% ≥ 1 CTC/30 ml threshold. CTC-positive cells were more frequent in HER2-negative tumors (p = 0.046). In patients younger than 50 years old, HER2-amplified and G1-G2 tumors had a higher possibility of being nondetectable CTCs. Heterogeneous expression of hormonal receptors (HRs) in samples from the same patients was found. Discordances between HR expression, HER2 and *TOP2A *status in CTCs and their primary tumor were found in the sequential blood samples. Less that 35% of patients switched their CTC status after receiving chemotherapy. EGFR-positive CTCs were associated with Luminal tumors (p = 0.03).

**Conclusions:**

This is the largest exploratory CTC biomarker analysis in nonmetastatic BC patients. Our study suggests that CTC biomarkers profiles might be useful as a surrogate marker for therapeutic selection and monitoring since heterogeneity of the biomarker distribution in CTCs and the lack of correlation with the primary tumor biomarker status were found. Further exploration of the association between EGFR-positive CTCs and Luminal tumors is warranted.

## Introduction

Breast cancer (BC) is the most frequently diagnosed malignancy in women [1]. Despite considerable advances in early detection, diagnosis, and treatment, BC is among the leading causes of cancer-related deaths in women because of recurrent metastatic disease.

Understanding the molecular profile of BC is becoming ever more relevant to patient care. Molecular subtypes were first described by Perou and colleagues [2,3], who mapped the phenotypic diversity to a specific gene expression pattern. An immunohistochemistry (IHC) profile based on the degree of expression of estrogen receptor (ER), progesterone receptor (PR), and human epithelial growth factor receptor 2 (HER2) similarly identifies subgroups of BC patients who will have similar gene expression patterns and clinical outcomes [3-5]. Subsequently, subgroups (within major groups) that have been defined as ER^-^, PR^-^, and HER2^- ^tumors that express cytokeratin (CK) 5/6 proteins or epidermal growth factor receptor (EGFR) or both represent another distinctive BC tumor subtype known as the core basal phenotype, which is associated with a worse prognosis [6]. Moreover, EGFR is considered essential in cancer cell migration and the intravasation process [7,8]. Therefore, we were interested in exploring the expression of EGFR in circulating tumor cells (CTCs) of patients with BC.

Subsequent studies showed differences in prognosis and differences in their response to therapeutic agents with respect to the subtype in specific cohorts of patients [4,5]. In addition to clinical and pathological factors currently used to guide prognosis and treatment, new evidence regarding the association of topoisomerase 2α (*TOP2A*) gene alterations and an increase in responsiveness to antracycline-containing regimens has been reported [9,10]. However, no studies have evaluated the *TOP2A *status in CTCs. These biomarker profiles do not guarantee a response to systemic therapy, and a fraction of patients will receive the established or investigational therapies without deriving any benefit. The presence of CTCS in the peripheral blood of non-metastatic BC patients has been associated with worse clinical outcomes [11-13]. In addition, there is increasing evidence of discrepancies between ER, PR, and HER2 expression in CTCs and the corresponding primary tumors, raising concern about the clinical implications of these observations[14-17]. Thus, the need to determine prognostically and therapeutically relevant markers in minimal residual disease is becoming important in order to increase personalized treatment options [18].

In this work, we sought to evaluate ER, PR, and EGFR expression and HER2 and TOP2A status in CTCs in a non-metastatic BC population. We further correlated the CTC findings with clinical and pathological characteristics of primary tumors and the distinct BC subtypes.

## Materials and methods

From March 2009 to September 2010, patients with stage I to IIIC BC were identified from the Breast Cancer Unit at the Hospital del Mar and Hospital Universitario de Jaén. The inclusion criteria were histological diagnosis of BC and availability of tissue for biomarker studies. Surgical procedure and systemic therapy were selected at the discretion of the treating physician with or without targeted therapy (namely, trastuzumab) for patients with HER2^+ ^BC. Medical charts of these patients were reviewed, and clinical details of these patients were included in a database.

A total of 98 patients donated three samples of 10 mL of blood at the time of first diagnosis. If adjuvant therapy (AT) was administered, post-treatment samples were obtained after three cycles of chemotherapy. If neoadjuvant therapy (NAT) was administered, samples were obtained at the end of treatment. For this study, we classified BC patients based on the pattern of expression of the hormone receptor (HR), estrogen and progesterone receptor, and HER2 status that identify three major distinct molecular BC subtypes [2,3]: luminal tumors, which are HR^+ ^and HER2^-^; HER2-amplified tumors; and those tumors that lack expression of the three receptors, known as triple-negative BC. This translational study was approved by the ethics review committees of the Hospital del Mar and Hospital de Jaén, and informed consent was obtained from all patients and healthy volunteers.

### Assessment of tumor biomarkers

Tumor specimens from archival tumor biopsies were available for HER2 and TOP2A status (*n *= 98 and *n *= 23), ER and PR (*n *= 98), p53 (*n *= 65), and Ki-67 (*n *= 98). ER and PR were routinely assessed by IHC by using 6F11 (diluted 1:40; Novocastra, Newcastle Upon Tyne, UK) and 312 (diluted 1:100) antibodies, respectively, in accordance with guidelines of the American Society of Clinical Oncology/College of American Pathologists [19]. Ki-67 proliferation index was assessed by using mouse monoclonal antibody MIB-1 (1:200 dilutions; Dako, Glostrup, Denmark), and the percentage of positively stained nuclei was calculated. Samples with any degree of p53 nuclear staining (clone DO-7; Novocastra Lab, Newcastle, UK) were considered positive. HER2 status was determined by IHC by using Herceptest (Dako) in all patients and was confirmed by fluorescence *in situ *hybridization (FISH) when indicated - Pathvysion HER2 DNA Probe Kit from Abbott Molecular (Abbott Park, IL, USA) in two centers and PharmaDX from Dako in two centers - in accordance with current recommendations [20]. *TOP2A *status was also evaluated by FISH by using a *TOP2A*/*CEP17 *FISH probe kit (Abbott Molecular Inc., Des Plaines, IL, USA). *TOP2A *amplification was considered if the *TOP2A*/*CEP17 *ratio was 2:1 or greater We considered polysomy 17 (p17) when cells had three or more copy numbers of centromeres for chromosome 17 per cell[21].

### Isolation and enumeration of circulating tumor cells

Blood (30 mL) was collected from each donor into three different blood collection tubes (CellSave Preservatives Tubes; Veridex, LLC, Raritan, NJ, USA), maintained at room temperature, and processed in parallel within a maximum of 72 hours after collection in accordance with the protocol established for our group [22]. Briefly, the samples were processed by density gradient centrifugation (Histopaque 1119; Sigma-Aldrich, St. Louis, MO, USA). For CTC enrichment, we used the Carcinoma Cell Enrichment and Detection kit with MACS technology (Miltenyi Biotec, Bergisch Gladbach, Germany). CTC enrichment was performed by selective immunomagnetic cell separation, using magnetic beads labeled with a multi-CK-specific antibody (CK3-11D5), which recognizes CK 7, 8, 18, and 19. CTCs were identified by immunocytochemical methods and visualized under a direct light microscope to perform the combined cytomorphological and immunophenotypic assessment. The cytomorphological criteria proposed by Meng and colleagues [23] (for example, high nuclear/cytoplasmic ratio and cells larger than white blood cells) were used to characterize a CK^+ ^cell as a CTC. As we analyzed three different tubes of 10 mL in each sample, we determined a case to be CTC^+ ^if at least one CTC was isolated in at least one of the three tubes. Therefore, patients were considered CTC^+ ^if at least one CTC^+ ^was captured in one tube of 10 ml of blood of the 30 ml of blood analyzed.

### Cell cultures and molecular biomarker assay feasibility

BC cell lines were obtained from the American Type Culture Collection (Manassas, VA, USA). In the analysis of recovery experiments, we analyzed control samples with high-level control numbers (2,000, 100, and 50 cells) from two human BC cell lines, MCF-7 and SKBR3, and with low level control numbers (10 and 5 cells and 1 cell) from four human BC cell lines: MCF-7, SKBR3, MDA-MB231, and T47D. Cells were spiked in 10 mL of venous blood from healthy volunteers, and control experiments were performed at least in triplicate. Cytospins were prepared afterward by density gradient centrifugation and immunomagnetic selection as per patient' samples. In our spiking experiments, recovery rates of tumor cells spiked into normal blood at the high-level control numbers were in the range of 40% to 60% and at the low level control numbers as shown in Supplementary Table S1 of Additional file 1. As negative controls, 16 blood samples from healthy volunteers without evidence of an epithelial malignancy were examined. Peripheral blood was drawn from the middle of vein puncture after the first 10-mL of blood were discarded. This precaution was undertaken in order to avoid contamination of the sample with epithelial cells from the skin during sample collection and to ensure a high specificity of the method. No CK^+ ^cells could be identified in these samples.

We next tested the technical feasibility of using, in assay, isolated CTCs that are more commonly used for biomarker assessment: protein expression by immunofluorescence (IF) and DNA amplification by FISH. Positive controls were created by using decreasing numbers of cells from BC cell lines spiked in blood from healthy volunteers, and cytospins were prepared as described above. For negative controls, blood from healthy volunteers was used and the primary antibody was omitted.

**Figure 1:**
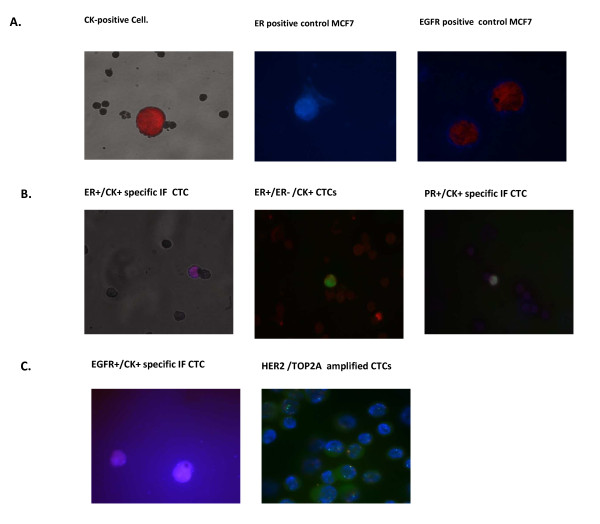
**Image galleries of representative biomarker profiles in CTCs**. **(a) **Image galleries after isolation, cytomorphological analysis, and detection of cytokeratin-positive (CK^+^) cells (red staining), estrogen receptor (ER), and epidermal growth factor receptor (EGFR) in MCF7 cell tumor lines. **(b) **Expression of different markers in patients with breast cancer through combination of stained CK^+ ^cells (red) with ER or progesterone receptor (PR) (blue). ER- and PR-specific immunofluorescence (IF) of circulating tumor cells (CTCs) was determined with Alexa 355. ER-specific IF of CTCs of a heterogeneous case is shown. **(c) **EFGR protein expression was detected by immunocytochemistry by using anti-human-EGFR antibodies (blue). EGFR-specific IF of CTCs was determined with Alexa 355. Human epidermal growth factor receptor 2 (HER2) and topoisomerase 2α (TOP2A) gene amplification was determined by fluorescence *in situ *hybridization (FISH) assay before immunophenotyping with anti-CK antibody (green). Image demonstrates HER2 amplification (red dots) and *TOP2A *amplification (green dots) compared to centromere 17 (CEP17; blue dots) in CTCs.

We tested whether ER, PR, and EGFR expression could be accurately determined by IF in CTCs by using the human BC cell line MCF-7; MDA-MB231 and SKBR3 as positive controls (Figure 1a). Using an anti-ER, -PR, and -EGFR antibody, we evaluated the expression seen in CTCs by the presence or absence of staining [24]. *HER2 *and *TOP2A *gene amplifications of BC cell lines were determined by FISH by using *HER2/TOP2A/CEP17 *probes. Tumor cells from SKBR3 (2,000, 100, and 50 cells) were spiked into whole blood, and cytospins were prepared under conditions identical to those of patient samples. Cells were analyzed for *HER2 *and *TOP2A *amplification according to standard criteria as described above for primary tumors. Absolute and relative copy numbers of *HER2 *and *TOP2A *genes in SKBR3 and MCF-7 cells after immunomagnetic separation from blood samples and fluorescence immunophenotyping and interphase cytogenetics as a tool for investigation of neoplasms (FICTION) analyses are shown in Supplementary Table S2 of Additional file 2.

### Characterization of ER, PR, and EGFR in circulating tumor cells

CK^+ ^and EGFR^+ ^cells were identified by IHC, and the signal was detected by chromogenic and fluorescent detection, respectively. CK-expressing cells were revealed by incubation with freshly prepared Fast Red TR/Naphthol AS-MX substrate solution (Sigma-Aldrich). Slides were washed once with phosphate-buffered saline and stained with Mayer's hematoxylin solution (Sigma-Aldrich). EGFR-expressing cells were revealed by incubation with primary monoclonal anti-human EGFR (Dako) diluted 1:25, followed by incubation with Alexa fluor 355 (Molecular Probes, now part of Invitrogen Corporation, Carlsbad, CA, USA). Epithelial tumor cells were identified and enumerated on the basis of their red staining for CK^+ ^cells and blue staining for EGFR^+ ^cells.

Slides positive for CK^+ ^cells were then stained with ER and PR rabbit anti-human primary antibodies and afterward with the corresponding anti-rabbit secondary antibodies labeled with Alexa fluor 488 for double- or triple-IF experiments following the laboratory requirements. Specific staining can easily be distinguished by the differential intracellular distribution of the examined molecules and the combination of direct and indirect IF in order to evaluate Ck^+^/ER and CK^+^/EGFR/PR (Figure 1b, c).

### Characterization of HER2 and *TOP2A *amplification in circulating tumor cells

*HER2 *and *TOP2A *amplification was determined by FISH. The *TOP2A/HER2*/*CEP17 *multi-color probe includes a *TOP2A *probe labeled with platinumBright495 (green), *HER2 *probe labeled with platinumBright550 (red), and chromosome enumeration probe *CEP17 *labeled with platinumBright415 (blue) (Kreatech, Durham, NC, USA) (Figure 1c).

After incubation, dehydratation, and air drying of cells, slides were co-denatured with the *TOP2A/HER2/CEP17 *multi-color probe for 5 minutes at 85°C. Hybridization with the probe previously denatured for 7 minutes at 75°C was performed at 37°C. After different washing steps, slides were counterstained with 4'-6-diamidino-2-phenylindole (DAPI) (Vector Laboratories, Burlingame, CA, USA). After FISH processing, CTCs were re-identified based on location and Ck^+ ^staining, and nuclei were scored for copies of *HER2*, *TOP2A*, and *CEP17 *as for primary tumors.

Identification and counting were done with a computerized fluorescence microscope (Zeiss AXIO Imager; Carl Zeiss, Jena, Germany). CK^+ ^cells were identified under a direct light microscope. After CK^+ ^cell detection in each tube independently, samples were brought to fluorescence light to evaluate cells with expression of CK^+^/EGFR/RP, CK^+^/ER, and CK^+^/*HER2 *and *TOP2A *status.

### Statistical methods

The main objectives were to investigate the status of five biomarkers in CTCs of patients with BC and to correlate this findings with clinical-pathological parameters and BC subtypes. Secondary objectives were to test changes in CTC count between inclusion and sequential samples, to evaluate the group of BC patients with non-detectable CTCs, and to evaluate the efficiency of processing three tubes with 10 mL of blood in terms of CTC count and biomarker assessment. The presence of at least one CTC per 10 mL was considered a positive result according to the reported analytic detection limit of our assay [22].

The statistical analysis was performed by using SPSS 14.0 software (SPSS Inc., Chicago, IL, USA). Data are expressed as means or numbers (percentages). Categorical variables were compared by Fisher exact test, and continuous variables were compared by Student *t *test. Two-tailed *P *values of less than 0.05 were considered statistically significant. Odds ratios (ORs) for the logistic model were calculated with their 95% confidence intervals (CIs) to assess the association between clinical-pathological variables and the CTC status.

## Results

### Detection of circulating tumor cells

Ninety-eight patients with BC were enrolled in our study. Before systemic treatment, we identified CTCs in 46 out of 98 patients (46.9%). For patients in whom CTCs were detected, the mean number of CTCs present was 3.4 cells per 30 mL of blood (range of 1 to 19). After three cycles of AT, CTCs were identified in 13 out of 38 patients (34.2%), and the mean value was 2.6 cells per 30 mL (range of 1 to 7). At the end of NAT, CTCs were detected in 16 out of 35 patients (45.7%). The mean CTC count was 2.6 cells per 30 mL in peripheral blood (range of 1 to 9). In a direct comparison of the incidence of CTC detection at baseline versus the sequential blood samples, no significant differences were found (*P *= 0.30 and *P *= 0.39, respectively).

**Figure 2:**
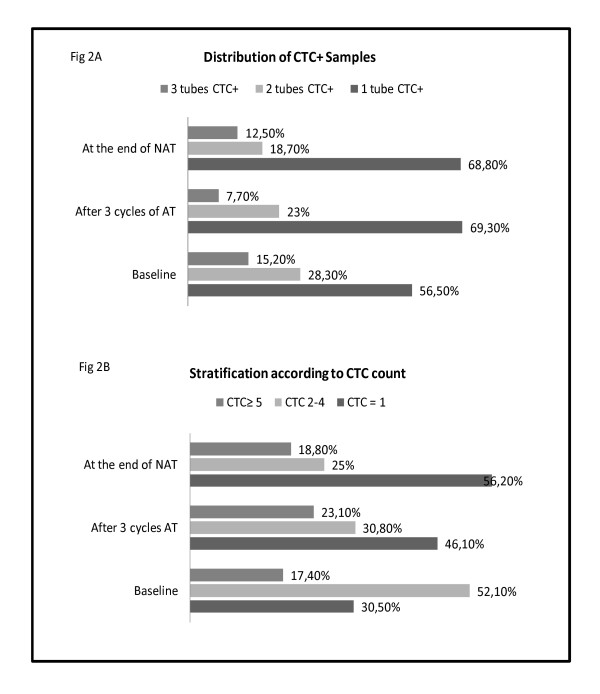
**Distribution of CTCs percentages and CTC count in samples**. **(a) **Distribution of circulating tumor cell-positive (CTC^+^) percentages in the three tubes collected at baseline and sequential blood samples. Dark gray: one tube is CTC^+^; medium gray: two tubes are CTC^+^; and light gray: three tubes are CTC^+^. **(b) **Stratification of CTC^+ ^patients according to the CTC count in three groups at baseline and sequential blood samples. Dark gray: at least five CTCs; medium gray: two to four CTCs; and light gray: one CTC. AT, adjuvant therapy; NAT, neoadjuvant therapy.

Distribution of CTC^+ ^samples in the three tubes collected and stratification according to the CTC count are shown in Figures 2a and 2b. In the baseline samples, in 56.5% of the patients, we were able to evaluate one of these phenotypes: CK^+^/ER, CK^+^/EGFR/RP, or CK^+^/HER2/*TOP2A*; in 28.3% of the patients, we were able to evaluate two of them. Although a large amount of blood was analyzed for each patient, we were able to evaluate the whole set of biomarkers in less than 16% of the patients at any time point. Additionally, in approximately 80% of CK^+ ^samples, four CTCs or fewer were captured to perform the biomarker analysis.

### Clinical-pathological characteristics and circulating tumor cell status

The patients' characteristics are consistent with those of an unselected early and locally advanced BC population. Main chemotherapy regimens included antracyclines (66.3%), taxanes (54.1%), cyclophosphamide (100%), and 5-fluorouracil (66.3%). Trastuzumab was administered only to patients with HER2^+ ^tumors (14.3%). Additionally, 72.4% of patients with HR^+ ^received endocrine therapy with letrozole (44.8%), tamoxifen (23.4%), or tamoxifen (2 to 3 years) followed by exemestane (4.1%). Clinical-pathological characteristics with stratification according to the baseline CTC status are described in Table 1. Three of the 14 patients with HER2-amplified primary tumors showed CTCs^+^, whereas in the remaining 11 patients with *HER2 *amplification, we did not detect CTCs (*P *= 0.046). In contrast, no significant correlation was found between CTC status and other clinical characteristics of patients.

**Table 1:** Circulating tumor cell status in relation to patient characteristics

	CTC^+^, number (percentage)	CTC^-^, number (percentage)	*P *value (χ^2^)
Age, years			
≤ 50	14 (41.2)	20 (58.8)	
> 50	32 (50)	32 (50)	NS
Histology			
Ductal	40 (47)	45 (53)	
Others	6 (46.2)	7 (53.8)	NS
Clinical tumor size			
≤ 2 cm	25 (54.3)	21 (45.7)	
> 2-5 cm	15 (39.5)	23 (60.5)	
> 5 cm	6 (42.9)	8 (57.1)	NS
Clinical nodal status			
cN0	28 (47.5)	31 (52.5)	
cN^+^	11 (34.4)	21 (65.6)	NS
Unknown	4 (57.1)	3 (42.9)	
Grade			
I	8 (38.1)	13 (61.9)	
II	15 (44.1)	19 (55.9)	
III	20 (55.5)	16 (44.5)	
Unknown	3 (42.9)	4 (57.1)	NS
Hormonal status			
HR^+^	35 (46.7)	40 (53.3)	
HR^-^	11 (47.8)	12 (52.2)	NS
HER2 status			
HER2^+^	3 (21.4)	11 (78.6)	
HER2^-^	43 (51.2)	41 (48.8)	0.046
p53 status			
p53^+^	26 (51)	25 (49)	
p53^-^	7 (50)	7 (50)	
Unknown	13 (39.4)	20 (60.6)	NS
Ki-67 percentage			
≤ 14%	20 (52.6)	18 (47.4)	
> 14%	26 (43.3)	34 (56.7)	NS

HR^+ ^indicates hormone receptor-positive: estrogen receptor-positive/progesterone receptor-positive (ER^+^/PR^+^), ER^+^/PR^-^, or ER^-^/PR^+^. HR^- ^indicates hormone receptor-negative. A total of 98 patients were included in this study. Information on clinical nodal status was available in 91 out of 98 (92.8%) patients and information on p53 status was available in 65 out of 98 (66.3%) patients at the time of study analysis. *P *values were determined by chi-squared tests. All statistical tests were two-sided. CTC, circulating tumor cell; HER2, human epidermal growth receptor 2; NS, not significant.

We attempted to look at the patients with undetectable CTCs in all of their analyzed blood samples and CTCs were not detectable in 43 (43.9%) patients. There was no statistical association between undetectable CTC status and tumor size, node status, histology, HR, Ki-67 and p53 status, or surgical procedures. However, we observed that a higher probability of patients with undetectable CTCs was recorded in patients who were younger than 50 years old (OR = 1.04, 95% CI = 1.01 to 10.8, *P *= 0.02) and had G1-G2 (OR = 6.9, 95% CI = 1.72 to 27.79, *P *= 0.006) or HER2-amplified (OR = 0.21, 95% CI = 0.05 to 0.84, *P *= 0.02) tumors.

### Hormonal status of circulating tumor cells and corresponding primary tumors

ER expression was evaluated before any systemic treatment, and ER staining was detected in 10 (50%) of the CK^+ ^CTC samples. Heterogeneity for ER expression was found in five of 10 (50%) patients with ER^+ ^CTCs; hence, ER^+ ^and ER^- ^CTCs were coexisting in the same sample. Among 13 patients with ER^+ ^tumors, CTCs detected before systemic therapy were analyzed for ER expression. As we expected, eight (61.5%) of these patients were classified as having ER^+ ^CTCs whereas five (38.5%) of them had ER^- ^CTCs.

PR status in CTCs was analyzed in 27 CTC^+ ^patients, and nine (33%) had significant nuclear PR expression. Heterogeneity for PR expression was found in one (11.1%) patient. When the PR expression was correlated between CTCs and their corresponding primary tumors, CTCs from 15 (68.2%) RP^+ ^primary tumors were classified as PR^- ^CTCs. In contrast, only seven (31.8%) patients with PR^+ ^tumors also had PR^+ ^CTCs. It is worth noting that there was no complete correlation between ER and PR expression in CTCs and their corresponding primary tumors, respectively (*P *= 0.17 and *P *= 0.55). Moreover, ER and PR status in CTCs did not show an association with clinical-pathological baseline characteristics of patients (data not shown).

### EGFR expression in circulating tumor cells

At baseline, EGFR^+ ^CTCs were detected in 27 (27.5%) patients. After three cycles and at the completion of chemotherapy, we detected EGFR^+ ^CTCs in five (13.9%) and four (11.1%) of the patients, respectively. No significant correlation was found between basal EGFR-CTC status and clinical-pathological characteristics of patients, including age, tumor size, nodal status, histology, nuclear tumor grade, p53 status, and Ki-67 and HER2 status. Remarkably, a higher proportion of patients with EGFR^+ ^CTCs was found in HR^+ ^patients (33.3% versus 8.7%, *P *= 0.01).

### HER2 and TOPO2A status of circulating tumor cells and corresponding primary tumors

Twenty-six CTC^+ ^patients were further evaluated for *ERBB2 *and *TOP2A *status by FISH in at least one CTC. Before systemic treatment, CTCs from three HER2^+ ^primary tumors were evaluated. It is of importance that none of them showed HER2 amplification in CTCs. Among patients with HER2^- ^tumors, 24 patients were classified as having HER2^- ^CTCs. Only one case with p17-CTCs was detected.

Among 26 CTC^+ ^patients analyzed, two cases showed *TOP2A-*amplified CTCs whereas the remaining patients had *TOP2A*^- ^CTCs. *TOP2A *status was available in 11 corresponding primary tumors. Among patients with *TOP2A*^- ^primary tumors, seven of them showed *TOP2A*^- ^CTCs whereas one had *TOP2A*-amplified CTCs and another had p17-CTCs. In contrast, three *TOP2A*-amplified primary tumors had *TOP2A*^- ^CTCs. Neither of the *TOP2A*-amplified CTC patients showed HER2 co-amplification. There was no association between HER2 or TOP2A CTC status and the corresponding primary tumor. In addition, baseline clinical-pathological characteristics were not linked to the HER2 and *TOP2A *status in CTCs. (data not shown).

### Circulating tumor cell characterization after a systemic treatment

After three cycles of AT, 13 patients (34.2%) had CTCs. In the biomarker analysis, ER expression in four out of six CTC^+ ^patients was found. Notably, one of the ER^- ^CTCs was detected in a patient who had an ER^+ ^primary tumor but no CTCs before systemic therapy. With regard to PR expression, significant PR staining was detected in three out of five CTC^+ ^patients. Interestingly, after three cycles of AT, one of the PR^+ ^CTC cases that came from a PR^+ ^tumor had PR^- ^CTCs at baseline. EGFR^+ ^CTCs were detected in five cases; one patient initially had EGFR^+ ^CTCs, whereas four (11.1%) cases were EGFR^- ^at baseline.

Significantly, 25 (69.4%) cases were still classified as having EGFR^- ^CTCs after three cycles of chemotherapy and six (16.6%) cases later were classified as having EGFR^- ^CTCs. In addition, HER2 status and *TOP2A *status were evaluated in seven CTC^+ ^patients. In six of them, HER2 and *TOP2A *genes were normal. Among patients with HER2^- ^tumors, discordant HER2 and *TOP2A *expression was found as one case was classified as having HER2 and *TOP2A*-co-amplified CTCs.

At the end of treatment, CTCs were detected in 17 (44.7%) of the patients. ER and PR expression of CTCs was detected in three patients (8.5%) and one patient (2.8%), respectively. Remarkably, two patients classified as having ER^- ^CTCs at the completion of NAT had ER^+ ^CTCs in the baseline sample and the corresponding primary tumor. In the 36 analyzed samples for EGFR expression, one case of EGFR^+ ^CTCs persisted whereas seven (19.4%) later were classified as having EGFR^- ^CTCs. Among cases that were initially classified as having EGFR^- ^CTCs, 25 (69.4%) were still negative for EGFR expression and three were classified as having EGFR^+ ^CTCs in the last blood sample.

In nine patients with CTCs still detectable, HER2 and *TOP2A *status was evaluated. Neither HER2 nor *TOP2A *amplification was found in any CTCs. Two of the corresponding primary tumors carried HER2 amplification, and seven were classified as HER2^- ^tumors.

**Figure 3:**
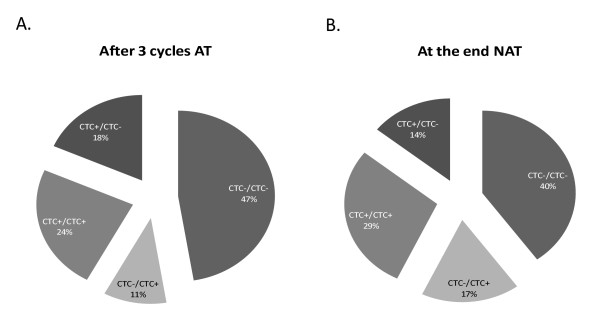
**Monitoring circulating tumor cell (CTC) status before and after systemic treatment**. Statuses are divided into four groups: (1) CTC^+^/CTC^+^: CTC^+ ^before and after treatment; (2) CTC^+^/CTC^-^: CTC^+ ^before but CTC^- ^after treatment; (3) CTC^-^/CTC^-^: CTC^- ^before and after treatment; and (4) CTC^-^/CTC^+^: CTC^- ^before but CTC^+ ^after systemic treatment. Pie charts show the percentages of patients in **(a) **the adjuvant therapy (AT) group and **(b) **the neoadjuvant therapy (NAT) group.

CTC status before and after systemic treatment is shown in Figure 3. It is notable that only around 30% of the patients switched their CTC status after systemic treatment. The predominant group of patients in the adjuvant and neoadjuvant setting is the group of patients with CTCs-negative before and after the systemic treatment.

### Breast cancer subtypes and circulating tumor cell biomarker profile

To address the clinically relevant need to identify subgroups of patients within luminal, *HER2*-amplified, and triple-negative tumors, we correlated biomarker expression in CTCs with the three major distinct molecular BC subtypes (Table 2). Note that there was a statistically significant association between only luminal tumors and higher risk of EFGR^+ ^CTCs (*P *= 0.03).

**Table 2:** Biomarker circulating tumor cell profile in relation to breast cancer subtypes

	Triple-negative number (percentage)	Luminal number (percentage)	HER2^+ ^number (percentage)	*P *value
ER^+ ^CTCs	1 (16.7)	7 (63.6)	2 (66.7)	NS
ER^- ^CTCs	5 (83.3)	4 (36.4)	1 (33.3)	
PR^+ ^CTCs	1(50)	8 (33.3)	0 (0)	NS
PR^- ^CTCs	1(50)	16 (66.7)	1 (100)	
EGFR^+ ^CTCs	2 (11.1)	24 (35.8)	1 (7.7)	0.03
EGFR^- ^CTCs	16 (88.9)	43 (64.2)	12 (92.3)	
HER2^+ ^CTCs	0 (0)	0 (0)^a^	0 (0)	Unable to determine
HER2^- ^CTCs	8 (100)	16 (100)	2 (100)	
TOP2A^+ ^CTCs	1 (12.5)	1 (6.3)^a^	0 (0)	NS
TOP2A^- ^CTCs	7 (87.5)	15 (93.7)	2 (100)	

^a^Polysomy 17-circulating tumor cell (17p CTC). The three breast cancer subtypes are triple-negative (estrogen receptor-negative [ER^-^], progesterone receptor-negative [PR^-^], and human epidermal growth factor receptor 2-negative [HER2^-^]), luminal (ER^+ ^and/or PR^+ ^and HER2^-^), and HER2 (*HER2*-amplified tumors). *P *values were determined by chi-squared tests. All statistical tests were two-sided. ER^+ ^CTCs, estrogen receptor-positive circulating tumor cells; ER^- ^CTCs, estrogen receptor-negative circulating tumor cells; PR^+ ^CTCs, progesterone receptor-positive circulating tumor cells; PR^- ^CTCs, progesterone receptor-negative circulating tumor cells; EGFR^+ ^CTCs, epidermal growth factor receptor-positive circulating tumor cells; EGFR^- ^CTCs, epidermal growth factor receptor-negative circulating tumor cells; HER2^+ ^CTCs, human epidermal growth factor receptor 2-positive circulating tumor cells; HER2^- ^CTCs, human epidermal growth factor receptor 2-negative circulating tumor cells; NS, not significant; TOP2A^+ ^CTCs, topoisomerase 2α-positive circulating tumor cells; TOP2A^- ^CTCs, topoisomerase 2α-negative circulating tumor cells.

## Discussion

To our knowledge, this study represents the largest analysis evaluating a set of five biomarkers in CTCs from sequential blood samples of patients with non-metastatic BC. In our work, we found a slightly higher detection rate in a similar amount of blood in comparison with those other studies using the CellSearch System (Veridex, LLC) before any treatment [13,25,26]. Besides, differences in the CTC count between the baseline samples and the post-treatment samples were not observed. However, owing to the small number of patients in each treatment group, our analysis was not sufficiently powered to draw any definitive conclusion in this endpoint.

Our observations indicate that phenotyping/genotyping analysis of CTCs is highly dependent on the detection rate in three different tubes and the low number of cells captured in the non-metastatic setting. Of note, the threshold of at least one CTC has been used before as a prognostic factor in patients with non-metastatic BC [12,13,26], reflecting that CellSearch shows similarly low CTCs counts in this setting. Optimization of CTC assays for high-throughput processing will be required to allow a comprehensive characterization of CTCs and large-scale clinical trials that use this emerging technology [27].

Several studies have addressed the correlation between CTC status and clinical-pathological parameters, but reported observations are still controversial. Lang and colleagues [25] found that CTCs were more frequently found in patients with HER2^+ ^tumors, whereas other researchers have not found any association between the CTC status and HER2^+ ^tumors [13,14]. In this study, CTC^+ ^cells were found more frequently in patients with HER2^- ^tumors, whereas other classic clinical-pathological parameters studied did not show a correlation with CTC status.

Non-detectable CTCs were observed in patients who were younger than 50 years old had primary tumors with *HER2 *amplification and G1-G2, and the higher risk was in the G1-G2 group. As in the metastatic setting [28], patients with poor prognostic factors had non-detectable CTCs. Such apparently contradictory results may be explained, in part, by the acquisition of mesenchymal antigens during the epithelial-mesenchymal transition (EMT) which facilitate the process of invasion and the metastatic cascade [29]. EMT-derived CTCs may have modulated their phenotype and acquired mesenchymal-like properties difficult to detect with the currently used detection methods [30]. As a result, patients with non-detectable CTCs and poor prognostic factors might represent a subset of patients with partial or complete EMT phenotype instead of an unequivocal undetectable CTC population.

Intriguingly, less than 35% of our population did not change their CTC status, suggesting that CTC population follows a pattern of neutral drift dynamics. This finding diverges with those of previous data [12,14] that reported a lower incidence of CTC detection rate after systemic treatment, especially in those patients who had received anti-HER2 therapies [14,31]. This discrepancy may be explained, at least in part, by the fact that we analyzed 30 mL instead of 7.5 mL and because only 14.3% of our patients received trastuzumab as part of their treatment.

In concordance with the study by Fehm and colleagues [17], our findings showed that ER and PR expression in CTCs were not correlated with ER or PR expression in the primary tumor. Heterogeneous CTC subpopulations with different HR phenotypes coexisting in the same blood sample were observed. Remarkably, an RNA-based method is not able to evaluate individual cells and detect heterogeneity among a CTC population, whereas the IF approach provides an additional biologically relevant characterization of different CTC subpopulations. Thus, it could be speculated that distinct HR expression in CTCs in the same patients might, in part, explain differences in response to both endocrine and chemotherapy treatments, although this association needs to be further characterized.

Changes of ER/PR phenotype or persistence of CTC phenotypes other than the primary tumor phenotype was also observed in our study in the samples after treatment. As all of the patients with sequential samples received chemotherapy, it cannot be excluded that drug-induced changes and clonal selection may be influenced by the interaction of CTCs with chemotherapy.

EGFR protein was expressed in 27% of CTCs at baseline and did not correlate with clinical and pathological parameters except for HR^+ ^tumors. Preclinical data have provided evidence that cross-talk between growth factor receptor (GFR) and ER pathway [32] may mediate the development of endocrine therapy resistance in HR^+ ^disease, although EGFR expression has been widely related to triple-negative BC tumors. The proposed biological mechanisms to explain how GFR signaling results in endocrine therapy resistance are conflicting [33-35]. Thus, we hypothesized that EGFR^+ ^CTCs might represent a potential negative biomarker of response to certain anti-cancer agents, including endocrine therapy in patients with HR^+ ^BC.

Besides, less than 25% of the EGFR^+ ^CTC patients became EGFR^- ^CTC after treatment, suggesting that conventional agents like chemotherapy or even trastuzumab eradicate partially EGFR^+ ^CTC subpopulations. We fully acknowledge that our results should be interpreted with caution because the sample size is limited and the small numbers of events limit our conclusions.

HER2 overexpression of CTCs in patients with BC has been well characterized in recent studies [14,15]. Discrepancies between HER2 status in CTCs and their corresponding primary tumors have been described in patients with early and metastatic BC [14,36]. In our study, the rate for HER2-amplified CTCs detected by FISH was null at baseline, which differs from the HER2^+ ^CTC rate reported by other groups using an IF approach. The lack of HER2^+ ^CTCs may be influenced by the fact that CTC populations are heterogeneous, and analyzing such a small number of CTCs may underestimate HER2^+ ^populations. Besides, CTCs with (2+) HER2 IF staining remain unresolved and could justify partially HER2^- ^CTCs. The optimal HER2 testing performance in CTCs has not been validated yet. Although IHC was the original method of assessment for HER2 status, IHC or IF alone cannot be recommended now for determining anti-HER2 treatment.

Previous studies have demonstrated that amplification of *TOP2A *in BC is not confined to those who are concomitantly HER2-amplified, suggesting that a proportion of HER2^- ^patients exhibit *TOP2A *alterations [9,37]. Our findings that two *TOP2A*-amplified CTCs come from HER2^- ^primary tumors and that the *HER2 *gene is not co-amplified in these CTCs are consistent with previous observations described in primary BC tissues [37,38].

After three cycles of AT, co-amplification of HER2 and *TOP2A *in CTCs was observed in one patient with HER2 and *TOP2A*^- ^tumor. This finding is consistent with a shift in tumor genotype and possibly dependency to alternative signaling pathways in HER2^- ^primary tumors. As all of the patients included in the sequential blood analysis received chemotherapy, it cannot be excluded that *HER2 *and *TOP2A *alterations in CTCs after treatment are influenced, at least in part, by the interaction with chemotherapy. According to other research groups, this finding opens up a window of opportunity because HER2^- ^BCs with co-amplification of *HER2 *and *TOP2A *in CTCs may exquisitely benefit anti-HER2 agents as well as antracycline-based regimens.

HER2^- ^CTCs were isolated in patients with either HER2^- ^or HER2^+ ^primary tumors after systemic treatment and trastuzumab therapy when recommended. This finding suggests that in HER2-amplified BC, HER2^- ^CTCs may have been selected by trastuzumab therapy. It is noteworthy that anti-HER2 agents are given in combination or sequentially [39,40] with chemotherapy, and the precise mechanism by which HER2^- ^CTCs persist is currently unknown.

Standard predictors for BC treatment selection are HR expression for endocrine therapy and HER2 status for anti-HER2 therapy [41]. Among the whole set of biomarkers evaluated in CTCs, only EFGR^+ ^CTCs were more frequent in luminal tumors compared with triple-negative and HER2-amplified tumors. It could be speculated that the association between EGFR^+ ^CTCs and luminal BC patients is explained, in part, by an increase of cancer cells expressing EGFR involved in the paracrine loop in which epidermal growth factor produced by tumor-associated macrophages increases the invasiveness and migration of BC cells that express EGFR [7,8], although EGFR expression has been widely related to lower HR levels, higher proliferation, genomic instability, and HER2 overexpression [42]. Although this association needs to be further characterized, luminal tumors might be more dependent than other BC subtypes on this mechanism that promotes cell migration and intravasation.

## Conclusions

Our findings exhibit the heterogeneity of biomarker distribution in CTCs and a lack of correlation with the primary tumor biomarker profile before and after chemotherapy. The lack of an association between HR, HER2, and TOP2A status in CTCs and BC subtypes may contribute to diversity in gene expression patterns and clinical outcomes within BC subtypes [43-45]. Biomarker characterization in CTCs might become a useful tool for selecting patients for tailored therapies and target drug development. However, these findings should be validated in a larger cohort of patients.

## Abbreviations

AT: adjuvant therapy; BC: breast cancer; CI: confidence interval; CK: cytokeratin; CTC: circulating tumor cell; EGFR: epidermal growth factor receptor; EMT: epithelial-mesenchymal transition; ER: estrogen receptor; FISH: fluorescence *in situ *hybridization; GFR: growth factor receptor; HR: hormone receptor; IF: immunofluorescence; IHC: immunohistochemistry; HER2: human epidermal growth factor receptor 2; NAT: neoadjuvant therapy; OR: odds ratio; p17: polysomy 17; PR: progesterone receptor; TOP2A: topoisomerase 2α.

## Competing interests

The authors declare that they have no competing interests.

## Authors' contributions

AF and LG helped to perform the immunomagnetic separation, the cell culture, and the immunofluorescence and FISH experiments. MJS helped to perform the immunomagnetic separation, the cell culture, and the immunofluorescence and FISH experiments and to draft the manuscript. RN helped to collect all of the blood samples and the clinical-pathological characteristics of the patients and to draft the manuscript. MM, PS-R, and MF helped to collect all of the blood samples and the clinical-pathological characteristics of the patients. MS, MR-R and JMC carried out the biomarker analysis in tissues. MD-R performed the statistical analysis. FS and JAL helped to draft the manuscript. All the authors participated in the design and coordination of the study. All the authors read and approved the final manuscript.

## Supplementary Material

Additional file 1 **Recovery rates with lower level control numbers (10, 5, 1 cells)**. Spiking experiments were performed in cultured cancer cell lines: MCF-7, SKBB3, MDA-MB 231 and T47D in triplicate. Recovery rate ranges from 33.3 to 66.6% at 1 cell level, from 33.3 to 66.6 at 5 cells level and from 53.3 to 73.3% at 10 cells level. Recovery data from single samples ranged from 15.2% to 173.2% because of the inherent variation in spiking of low numbers of cells. However, all spiked samples levels regardless of the low number added, had detectable cells except in one sample of the level of 1 cell from the SKBR3 cell line. Click here for file

Additional file 2 **Absolute and relative copy numbers of *HER-2 *and *TOP2A *genes in SKBR3, MCF7 cells, after immunomagnetic separation from blood samples and FICTION analyses**. Click here for file

## References

[B1] Jemal A Siegel R Xu J Ward E Cancer statistics, 2010 CA Cancer J Clin 2010 60 277 300 10.3322/caac.20073 20610543

[B2] Perou CM Sorlie T Eisen MB van de Rijn M Jeffrey SS Rees CA Pollack JR Ross DT Johnsen H Akslen LA Fluge O Pergamenschikov A Williams C Zhu SX Lønning PE Børresen-Dale AL Brown PO Botstein D Molecular portraits of human breast tumours Nature 2000 406 747 752 10.1038/35021093 10963602

[B3] Sorlie T Perou CM Tibshirani R Aas T Geisler S Johnsen H Hastie T Eisen MB van de Rijn M Jeffrey SS Thorsen T Quist H Matese JC Brown PO Botstein D Lønning PE Børresen-Dale AL Gene expression patterns of breast carcinomas distinguish tumor subclasses with clinical implications Proc Natl Acad Sci USA 2001 98 10869 10874 10.1073/pnas.191367098 11553815 PMC58566

[B4] Carey LA Dees EC Sawyer L Gatti L Moore DT Collichio F Ollila DW Sartor CI Graham ML Perou CM The triple negative paradox: primary tumor chemosensitivity of breast cancer subtypes Clin Cancer Res 2007 13 2329 2334 10.1158/1078-0432.CCR-06-1109 17438091

[B5] Hugh J Hanson J Cheang MC Nielsen TO Perou CM Dumontet C Reed J Krajewska M Treilleux I Rupin M Magherini E Mackey J Martin M Vogel C Breast cancer subtypes and response to docetaxel in node-positive breast cancer: use of an immunohistochemical definition in the BCIRG 001 trial J Clin Oncol 2009 27 1168 1176 10.1200/JCO.2008.18.1024 19204205 PMC2667821

[B6] Cheang MC Voduc D Bajdik C Leung S McKinney S Chia SK Perou CM Nielsen TO Basal-like breast cancer defined by five biomarkers has superior prognostic value than triple-negative phenotype Clin Cancer Res 2008 14 1368 1376 10.1158/1078-0432.CCR-07-1658 18316557

[B7] Wyckoff J Wang W Lin EY Wang Y Pixley F Stanley ER Graf T Pollard JW Segall J Condeelis J A paracrine loop between tumor cells and macrophages is required for tumor cell migration in mammary tumors Cancer Res 2004 64 7022 7029 10.1158/0008-5472.CAN-04-1449 15466195

[B8] Goswami S Sahai E Wyckoff JB Cammer M Cox D Pixley FJ Stanley ER Segall JE Condeelis JS Macrophages promote the invasion of breast carcinoma cells via a colony-stimulating factor-1/epidermal growth factor paracrine loop Cancer Res 2005 65 5278 5283 10.1158/0008-5472.CAN-04-1853 15958574

[B9] O'Malley FP Chia S Tu D Shepherd LE Levine MN Bramwell VH Andrulis IL Pritchard KI Topoisomerase II alpha and responsiveness of breast cancer to adjuvant chemotherapy J Natl Cancer Inst 2009 101 644 650 10.1093/jnci/djp067 19401546 PMC2677575

[B10] Ejlertsen B Jensen MB Nielsen KV Balslev E Rasmussen BB Willemoe GL Hertel PB Knoop AS Mouridsen HT Brunner N HER2, TOP2A, and TIMP-1 and responsiveness to adjuvant anthracycline-containing chemotherapy in high-risk breast cancer patients J Clin Oncol 2010 28 984 990 10.1200/JCO.2009.24.1166 20038724

[B11] Saloustros E Perraki M Apostolaki S Kallergi G Xyrafas A Kalbakis K Agelaki S Kalykaki A Georgoulias V Mavroudis D Cytokeratin-19 mRNA-positive circulating tumor cells during follow-up of patients with operable breast cancer: prognostic relevance for late relapse Breast Cancer Res 2011 13 R60 10.1186/bcr2897 21663668 PMC3218949

[B12] Pierga JY Bidard FC Mathiot C Brain E Delaloge S Giachetti S de Cremoux P Salmon R Vincent-Salomon A Marty M Circulating tumor cell detection predicts early metastatic relapse after neoadjuvant chemotherapy in large operable and locally advanced breast cancer in a phase II randomized trial Clin Cancer Res 2008 14 7004 7010 10.1158/1078-0432.CCR-08-0030 18980996

[B13] Rack BK Schindlbeck C Andergassen U Schneeweiss A Zwingers T Lichtenegger W Beckmann M Sommer HL Pantel K Janni W Use of circulating tumor cells (CTC) in peripheral blood of breast cancer patients before and after adjuvant chemotherapy to predict risk for relapse: the SUCCES trial (abstract) J Clin Oncol 2010 28 15 Suppl a1300

[B14] Riethdorf S Muller V Zhang L Rau T Loibl S Komor M Roller M Huober J Fehm T Schrader I Hilfrich J Holms F Tesch H Eidtmann H Untch M von Minckwitz G Pantel K Detection and HER2 expression of circulating tumor cells: prospective monitoring in breast cancer patients treated in the neoadjuvant GeparQuattro trial Clin Cancer Res 2010 16 2634 2645 10.1158/1078-0432.CCR-09-2042 20406831

[B15] Wulfing P Borchard J Buerger H Heidl S Zanker KS Kiesel L Brandt B HER2-positive circulating tumor cells indicate poor clinical outcome in stage I to III breast cancer patients Clin Cancer Res 2006 12 1715 1720 10.1158/1078-0432.CCR-05-2087 16551854

[B16] Ignatiadis M Rothe F Chaboteaux C Durbecq V Rouas G Criscitiello C Metallo J Kheddoumi N Singhal SK Michiels S Veys I Rossari J Larsimont D Carly B Pestrin M Bessi S Buxant F Liebens F Piccart M Sotiriou C HER2-positive circulating tumor cells in breast cancer PLoS One 2011 6 e15624 10.1371/journal.pone.0015624 21264346 PMC3018524

[B17] Fehm T Hoffmann O Aktas B Becker S Solomayer EF Wallwiener D Kimmig R Kasimir-Bauer S Detection and characterization of circulating tumor cells in blood of primary breast cancer patients by RT-PCR and comparison to status of bone marrow disseminated cells Breast Cancer Res 2009 11 R59 10.1186/bcr2349 19664291 PMC2750121

[B18] Mego M Mani SA Cristofanilli M Molecular mechanisms of metastasis in breast cancer--clinical applications Nat Rev Clin Oncol 2010 7 693 701 10.1038/nrclinonc.2010.171 20956980

[B19] Hammond ME Hayes DF Dowsett M Allred DC Hagerty KL Badve S Fitzgibbons PL Francis G Goldstein NS Hayes M Hicks DG Lester S Love R Mangu PB McShane L Miller K Osborne CK Paik S Perlmutter J Rhodes A Sasano H Schwartz JN Sweep FC Taube S Torlakovic EE Valenstein P Viale G Visscher D Wheeler T Williams RB American Society of Clinical Oncology/College Of American Pathologists guideline recommendations for immunohistochemical testing of estrogen and progesterone receptors in breast cancer J Clin Oncol 2010 28 2784 2795 10.1200/JCO.2009.25.6529 20404251 PMC2881855

[B20] Sauter G Lee J Bartlett JM Slamon DJ Press MF Guidelines for human epidermal growth factor receptor 2 testing: biologic and methodologic considerations J Clin Oncol 2009 27 1323 1333 10.1200/JCO.2007.14.8197 19204209

[B21] Salido M Tusquets I Corominas JM Suarez M Espinet B Corzo C Bellet M Fabregat X Serrano S Sole F Polysomy of chromosome 17 in breast cancer tumors showing an overexpression of ERBB2: a study of 175 cases using fluorescence *in situ *hybridization and immunohistochemistry Breast Cancer Res 2005 7 R267 273 10.1186/bcr996 15743507 PMC1064140

[B22] Gaforio JJ Serrano MJ Sanchez-Rovira P Sirvent A Delgado-Rodriguez M Campos M de la Torre N Algarra I Duenas R Lozano A Detection of breast cancer cells in the peripheral blood is positively correlated with estrogen-receptor status and predicts for poor prognosis Int J Cancer 2003 107 984 990 10.1002/ijc.11479 14601059

[B23] Meng S Tripathy D Frenkel EP Shete S Naftalis EZ Huth JF Beitsch PD Leitch M Hoover S Euhus D Haley B Morrison L Fleming TP Herlyn D Terstappen LW Fehm T Tucker TF Lane N Wang J Uhr JW Circulating tumor cells in patients with breast cancer dormancy Clin Cancer Res 2004 10 8152 8162 10.1158/1078-0432.CCR-04-1110 15623589

[B24] Payne RE Yague E Slade MJ Apostolopoulos C Jiao LR Ward B Coombes RC Stebbing J Measurements of EGFR expression on circulating tumor cells are reproducible over time in metastatic breast cancer patients Pharmacogenomics 2009 10 51 57 10.2217/14622416.10.1.51 19102715

[B25] Lang JE Mosalpuria K Cristofanilli M Krishnamurthy S Reuben J Singh B Bedrosian I Meric-Bernstam F Lucci A HER2 status predicts the presence of circulating tumor cells in patients with operable breast cancer Breast Cancer Res Treat 2009 113 501 507 10.1007/s10549-008-9951-2 18327638 PMC5847290

[B26] Bidard FC Mathiot C Delaloge S Brain E Giachetti S de Cremoux P Marty M Pierga JY Single circulating tumor cell detection and overall survival in nonmetastatic breast cancer Ann Oncol 2010 21 729 733 10.1093/annonc/mdp391 19850639

[B27] Punnoose EA Atwal SK Spoerke JM Savage H Pandita A Yeh RF Pirzkall A Fine BM Amler LC Chen DS Lackner MR Molecular biomarker analyses using circulating tumor cells PLoS One 2010 5 e12517 10.1371/journal.pone.0012517 20838621 PMC2935889

[B28] Mego M De Giorgi U Dawood S Wang X Valero V Andreopoulou E Handy B Ueno NT Reuben JM Cristofanilli M Characterization of metastatic breast cancer patients with nondetectable circulating tumor cells Int J Cancer 2011 129 417 423 10.1002/ijc.25690 20857493

[B29] Yang J Weinberg RA Epithelial-mesenchymal transition: at the crossroads of development and tumor metastasis Dev Cell 2008 14 818 829 10.1016/j.devcel.2008.05.009 18539112

[B30] Kallergi G Papadaki MA Politaki E Mavroudis D Georgoulias V Agelaki S Epithelial to mesenchymal transition markers expressed in circulating tumour cells of early and metastatic breast cancer patients Breast Cancer Res 2011 13 R59 10.1186/bcr2896 21663619 PMC3218948

[B31] Pierga JY Hajage D Bachelot T Delaloge S Brain E Campone M Dieras V Rolland E Mignot L Mathiot C Bidard FC High independent prognostic and predictive value of circulating tumor cells compared with serum tumor markers in a large prospective trial in first-line chemotherapy for metastatic breast cancer patients Ann Oncol 2012 23 618 624 10.1093/annonc/mdr263 21642515

[B32] Shou J Massarweh S Osborne CK Wakeling AE Ali S Weiss H Schiff R Mechanisms of tamoxifen resistance: increased estrogen receptor-HER2/neu cross-talk in ER/HER2-positive breast cancer J Natl Cancer Inst 2004 96 926 935 10.1093/jnci/djh166 15199112

[B33] Osborne CK Schiff R Growth factor receptor cross-talk with estrogen receptor as a mechanism for tamoxifen resistance in breast cancer Breast 2003 12 362 367 10.1016/S0960-9776(03)00137-1 14659106

[B34] Font de Mora J Brown M AIB1 is a conduit for kinase-mediated growth factor signaling to the estrogen receptor Mol Cell Biol 2000 20 5041 5047 10.1128/MCB.20.14.5041-5047.2000 10866661 PMC85954

[B35] Creighton CJ Massarweh S Huang S Tsimelzon A Hilsenbeck SG Osborne CK Shou J Malorni L Schiff R Development of resistance to targeted therapies transforms the clinically associated molecular profile subtype of breast tumor xenografts Cancer Res 2008 68 7493 7501 10.1158/0008-5472.CAN-08-1404 18794137 PMC2556890

[B36] Meng S Tripathy D Shete S Ashfaq R Haley B Perkins S Beitsch P Khan A Euhus D Osborne C Frenkel E Hoover S Leitch M Clifford E Vitetta E Morrison L Herlyn D Terstappen LW Fleming T Fehm T Tucker T Lane N Wang J Uhr J HER-2 gene amplification can be acquired as breast cancer progresses Proc Natl Acad Sci USA 2004 101 9393 9398 10.1073/pnas.0402993101 15194824 PMC438987

[B37] Glynn RW Mahon S Curran C Callagy G Miller N Kerin MJ TOP2A amplification in the absence of that of HER-2/neu: toward individualization of chemotherapeutic practice in breast cancer Oncologist 2011 16 949 955 10.1634/theoncologist.2011-0071 21705665 PMC3228145

[B38] Nielsen KV Muller S Moller S Schonau A Balslev E Knoop AS Ejlertsen B Aberrations of ERBB2 and TOP2A genes in breast cancer Mol Oncol 2010 4 161 168 10.1016/j.molonc.2009.11.001 19945923 PMC5527893

[B39] Gianni L Eiermann W Semiglazov V Manikhas A Lluch A Tjulandin S Zambetti M Vazquez F Byakhow M Lichinitser M Climent MA Ciruelos E Ojeda B Mansutti M Bozhok A Baronio R Feyereislova A Barton C Valagussa P Baselga J Neoadjuvant chemotherapy with trastuzumab followed by adjuvant trastuzumab versus neoadjuvant chemotherapy alone, in patients with HER2-positive locally advanced breast cancer (the NOAH trial): a randomised controlled superiority trial with a parallel HER2-negative cohort Lancet 2010 375 377 384 10.1016/S0140-6736(09)61964-4 20113825

[B40] Piccart-Gebhart MJ Procter M Leyland-Jones B Goldhirsch A Untch M Smith I Gianni L Baselga J Bell R Jackisch C Cameron D Dowsett M Barrios CH Steger G Huang CS Andersson M Inbar M Lichinitser M Láng I Nitz U Iwata H Thomssen C Lohrisch C Suter TM Rüschoff J Suto T Greatorex V Ward C Straehle C McFadden E Trastuzumab after adjuvant chemotherapy in HER2-positive breast cancer N Engl J Med 2005 353 1659 1672 10.1056/NEJMoa052306 16236737

[B41] Di Cosimo S Baselga J Management of breast cancer with targeted agents: importance of heterogeneity. [corrected] Nat Rev Clin Oncol 2010 7 139 147 10.1038/nrclinonc.2009.234 20125090

[B42] Rimawi MF Shetty PB Weiss HL Schiff R Osborne CK Chamness GC Elledge RM Epidermal growth factor receptor expression in breast cancer association with biologic phenotype and clinical outcomes Cancer 2010 116 1234 1242 10.1002/cncr.24816 20082448 PMC2829330

[B43] Yang XR Chang-Claude J Goode EL Couch FJ Nevanlinna H Milne RL Gaudet M Schmidt MK Broeks A Cox A Cox A Fasching PA Hein R Spurdle AB Blows F Driver K Flesch-Janys D Heinz J Sinn P Vrieling A Heikkinen T Aittomäki K Heikkilä P Blomqvist C Lissowska J Peplonska B Chanock S Figueroa J Brinton L Hall P Czene K Associations of breast cancer risk factors with tumor subtypes: a pooled analysis from the Breast Cancer Association Consortium studies J Natl Cancer Inst 2011 103 250 263 10.1093/jnci/djq526 21191117 PMC3107570

[B44] Blows FM Driver KE Schmidt MK Broeks A van Leeuwen FE Wesseling J Cheang MC Gelmon K Nielsen TO Blomqvist C Heikkilä P Heikkinen T Nevanlinna H Akslen LA Bégin LR Foulkes WD Couch FJ Wang X Cafourek V Olson JE Baglietto L Giles GG Severi G McLean CA Southey MC Rakha E Green AR Ellis IO Sherman ME Lissowska J Subtyping of breast cancer by immunohistochemistry to investigate a relationship between subtype and short and long term survival: a collaborative analysis of data for 10,159 cases from 12 studies PLoS Med 2010 7 e1000279 10.1371/journal.pmed.1000279 20520800 PMC2876119

[B45] Staaf J Ringner M Vallon-Christersson J Jonsson G Bendahl PO Holm K Arason A Gunnarsson H Hegardt C Agnarsson BA Luts L Grabau D Fernö M Malmström PO Johannsson OT Loman N Barkardottir RB Borg A Identification of subtypes in human epidermal growth factor receptor 2--positive breast cancer reveals a gene signature prognostic of outcome J Clin Oncol 2010 28 1813 1820 10.1200/JCO.2009.22.8775 20231686

