# Supplementary material for: Biomarkers characterization of circulating tumour cells in breast cancer patients
Source: Breast Cancer Res. 2012 May 3;14(3):R71. doi: 10.1186/bcr3180 (PMC3446333; doi:10.1186/bcr3180)
Supplement: Additional file 1 — Recovery rates with lower level control numbers (10, 5, 1 cells). Spiking experiments were performed in cultured cancer cell lines: MCF-7, SKBB3, MDA-MB 231 and T47D in triplicate. Recovery rate ranges from 33.3 to 66.6% at 1 cell level, from 33.3 to 66.6 at 5 cells level and from 53.3 to 73.3% at 10 cells level. Recovery data from single samples ranged from 15.2% to 173.2% because of the inherent variation in spiking of low numbers of cells. However, all spiked samples levels regardless of the low number added, had detectable cells except in one sample of the level of 1 cell from the SKBR3 cell line. [file bcr3180-S1.PDF]

**Supplementary Table 1**

**Precision data for cell lines controls in low level control numbers**

| Cell Lines | Number of cells (n) | Number of repeats (n) | Recovery mean ± SD | Recovery mean (%) | Coefficient Variability (%) |
|---|---|---|---|---|---|
| **MCF-7** | 1 | 3 | 0.33 ± 0.57 | 33.3 | 173.2 |
| | 5 | 3 | 1.6 ± 1.5 | 33.3 | 91.6 |
| | 10 | 3 | 6 ± 1.7 | 60 | 28.9 |
| **SKBR3** | 1 | 3 | - | - | - |
| | 5 | 3 | 2.3 ± 1.5 | 46.6 | 49.4 |
| | 10 | 3 | 5.3 ± 1.5 | 53.5 | 28.6 |
| **MDA-MB231** | 1 | 3 | 0.3 ± 0.5 | 33.3 | 173.2 |
| | 5 | 3 | 2.6 ± 0.5 | 53.3 | 21.7 |
| | 10 | 3 | 5.6 ± 1.1 | 56.6 | 20.3 |
| **T47D** | 1 | 3 | 0.6 ± 0.5 | 66 | 86.6 |
| | 5 | 3 | 3.3 ± 0.5 | 66 | 17.3 |
| | 10 | 3 | 7.3 ± 1.1 | 73.3 | 15.2 |
