# Supplementary material for: Biomarkers characterization of circulating tumour cells in breast cancer patients
Source: Breast Cancer Res. 2012 May 3;14(3):R71. doi: 10.1186/bcr3180 (PMC3446333; doi:10.1186/bcr3180)
Supplement: Additional file 2 — Absolute and relative copy numbers of HER-2 and TOP2A genes in SKBR3, MCF7 cells, after immunomagnetic separation from blood samples and FICTION analyses. [file bcr3180-S2.PDF]

Supplementary Table 2.

	HER-2 copy number		*TOP2A* copy number	
**Cell line**	**Absolute (mean ± SD)**	**Relative to 17 centromere**	**Absolute (mean ± SD)**	**Relative to 17 centromere**
**Leukocytes**[a]	2.00 ± 0.00	1.00	2.00 ± 0.00	1.00
**SKBR3**	25.87 ± 7.39	3.81[b]	9.48 ± 2.55	1.40[b]
**MCF7**	2.02 ± 0.24	0.66[c]	2.01 ± 0.26	0.66[c]

[a]Samples internal control (400 cells). [b]Gene amplification. [c]Gene deletion.
